# Supplementary material for: OpenMM 7: Rapid development of high performance algorithms for molecular dynamics
Source: PLoS Comput Biol. 2017 Jul 26;13(7):e1005659. doi: 10.1371/journal.pcbi.1005659 (PMC5549999; doi:10.1371/journal.pcbi.1005659)
Supplement: S1 Source Code — (ZIP) [file pcbi.1005659.s002.zip › OpenMM-7.0.1-Source/docs/api-python/generated/simtk.openmm.openmm.AmoebaPiTorsionForce.html]

AmoebaPiTorsionForce — OpenMM 7.0.1.dev-5e86c4f documentation


# AmoebaPiTorsionForce¶

*class* `simtk.openmm.openmm.``AmoebaPiTorsionForce`(*\*args*)¶
:   This class implements the Amoeba pi-torsion interaction.

    To use it, create an AmoebaPiTorsionForce object then call addPiTorsion() once for each torsion. After a torsion has been added, you can modify its force field parameters by calling setPiTorsionParameters(). This will have no effect on Contexts that already exist unless you call updateParametersInContext().

    `__init__`(*self*) → AmoebaPiTorsionForce¶
    :   \_\_init\_\_(self, other) -> AmoebaPiTorsionForce

        Create an AmoebaPiTorsionForce.

    Methods

    |  |  |
    | --- | --- |
    | `__init__`((self) -> AmoebaPiTorsionForce) | \_\_init\_\_(self, other) -> AmoebaPiTorsionForce |
    | `addPiTorsion`((self, particle1, particle2, ...) | Add a torsion term to the force field. |
    | `getForceGroup`((self) -> int) | Get the force group this Force belongs to. |
    | `getNumPiTorsions`((self) -> int) | Get the number of pi torsion terms in the potential function |
    | `getPiTorsionParameters`(self, index) | Get the force field parameters for a torsion term. |
    | `setForceGroup`(self, group) | Set the force group this Force belongs to. |
    | `setPiTorsionParameters`(self, index, ...) | Set the force field parameters for a pi torsion term. |
    | `updateParametersInContext`(self, context) | Update the per-torsion parameters in a Context to match those stored in this Force object. |
    | `usesPeriodicBoundaryConditions`((self) -> bool) | Returns whether or not this force makes use of periodic boundary conditions. |

    `getNumPiTorsions`(*self*) → int¶
    :   Get the number of pi torsion terms in the potential function

    `addPiTorsion`(*self*, *particle1*, *particle2*, *particle3*, *particle4*, *particle5*, *particle6*, *k*) → int¶
    :   Add a torsion term to the force field.

        |  |  |
        | --- | --- |
        | Parameters: | - **particle1** (*int*) – the index of the first particle connected by the torsion - **particle2** (*int*) – the index of the second particle connected by the torsion - **particle3** (*int*) – the index of the third particle connected by the torsion - **particle4** (*int*) – the index of the fourth particle connected by the torsion - **particle5** (*int*) – the index of the fifth particle connected by the torsion - **particle6** (*int*) – the index of the sixth particle connected by the torsion - **k** (*double*) – the force constant for the torsion |
        | Returns: | the index of the torsion that was added |
        | Return type: | int |

    `getPiTorsionParameters`(*self*, *index*)¶
    :   Get the force field parameters for a torsion term.

        |  |  |
        | --- | --- |
        | Parameters: | **index** (*int*) – the index of the torsion for which to get parameters |
        | Returns: | - **particle1** (*int*) –   the index of the first particle connected by the torsion - **particle2** (*int*) –   the index of the second particle connected by the torsion - **particle3** (*int*) –   the index of the third particle connected by the torsion - **particle4** (*int*) –   the index of the fourth particle connected by the torsion - **particle5** (*int*) –   the index of the fifth particle connected by the torsion - **particle6** (*int*) –   the index of the sixth particle connected by the torsion - **k** (*double*) –   the force constant for the torsion |

    `setPiTorsionParameters`(*self*, *index*, *particle1*, *particle2*, *particle3*, *particle4*, *particle5*, *particle6*, *k*)¶
    :   Set the force field parameters for a pi torsion term.

        |  |  |
        | --- | --- |
        | Parameters: | - **index** (*int*) – the index of the torsion for which to set parameters - **particle1** (*int*) – the index of the first particle connected by the torsion - **particle2** (*int*) – the index of the second particle connected by the torsion - **particle3** (*int*) – the index of the third particle connected by the torsion - **particle4** (*int*) – the index of the fourth particle connected by the torsion - **particle5** (*int*) – the index of the fifth particle connected by the torsion - **particle6** (*int*) – the index of the sixth particle connected by the torsion - **k** (*double*) – the force constant for the torsion |

    `updateParametersInContext`(*self*, *context*)¶
    :   Update the per-torsion parameters in a Context to match those stored in this Force object. This method provides an efficient method to update certain parameters in an existing Context without needing to reinitialize it. Simply call setPiTorsionParameters() to modify this object’s parameters, then call updateParametersInContext() to copy them over to the Context.

        The only information this method updates is the values of per-torsion parameters. The set of particles involved in a torsion cannot be changed, nor can new torsions be added.

    `usesPeriodicBoundaryConditions`(*self*) → bool¶
    :   Returns whether or not this force makes use of periodic boundary conditions.

        |  |  |
        | --- | --- |
        | Returns: | true if nonbondedMethod uses PBC and false otherwise |
        | Return type: | bool |

    `__delattr__`¶
    :   x.\_\_delattr\_\_(‘name’) <==> del x.name

    `__format__`()¶
    :   default object formatter

    `__getattribute__`¶
    :   x.\_\_getattribute\_\_(‘name’) <==> x.name

    `__hash__`¶

    `__reduce__`()¶
    :   helper for pickle

    `__reduce_ex__`()¶
    :   helper for pickle

    `__sizeof__`() → int¶
    :   size of object in memory, in bytes

    `__str__`¶

    `getForceGroup`(*self*) → int¶
    :   Get the force group this Force belongs to.

    `setForceGroup`(*self*, *group*)¶
    :   Set the force group this Force belongs to.

        |  |  |
        | --- | --- |
        | Parameters: | **group** (*int*) – the group index. Legal values are between 0 and 31 (inclusive). |

High performance molecular simulation on GPUs


### Navigation

- Application Layer
- Library Layer
  - Core Objects
  - Forces
  - Integrators
  - Extras

©2015, Stanford University and the Authors.
|
Powered by Sphinx 1.3.1
& Alabaster 0.7.7
|
Page source
